# Attitudes of dermatologists in the southeastern United States regarding treatment of alopecia areata: a cross-sectional survey study

**DOI:** 10.1186/1471-5945-9-11

**Published:** 2009-11-12

**Authors:** Niyati Mukherjee, Dean S Morrell, Madeleine Duvic, Paul W Stewart, Lowell A Goldsmith

**Affiliations:** 1 Department of Internal Medicine, University of North Carolina Hospitals, Chapel Hill, NC, USA; 2 Department of Dermatology, University of North Carolina School of Medicine, Chapel Hill, NC, USA; 3 Department of Dermatology, University of Texas, MD Anderson Cancer Center, Houston, TX, USA; 4 Department of Biostatistics, University of North Carolina School of Public Health, Chapel Hill, NC, USA; 5 Department of Dermatology, University of North Carolina School of Medicine, Chapel Hill, NC, USA

## Abstract

**Background:**

Little evidence exists to guide treatment of alopecia areata (AA). The current practices in treatment of children compared to adults and of progressive stages of hair loss are unknown. The objective of this study was to examine the current practices of southeastern United States dermatologists for the treatment of AA.

**Methods:**

Dermatologists were sent anonymous questionnaires regarding their treatment practices by mail. Respondents' frequencies of treatment in children compared to adults and in patchy hair loss compared to widespread hair loss were compared with Wilcoxon signed-ranks tests and Friedman tests. As a secondary source, the National Alopecia Areata Registry (NAAR) database was analyzed for patients' treatment histories.

**Results:**

Survey results suggested that dermatologists recommend treatment less frequently for children than adults and for more advanced hair loss. NAAR data confirmed that offering no treatment for AA is relatively common.

**Conclusion:**

Dermatologists' treatment of AA is inconsistent. A stronger evidence base will provide more consistent treatment options.

## Background

Alopecia areata (AA) is relatively common, accounting for 2% of new dermatology outpatient visits in the United States and the United Kingdom [1], but little is yet known about optimal treatment of the disease. Many AA treatments, including topical, intralesional and oral corticosteroids, minoxidil, contact sensitizers, anthralin, and PUVA [2], have not been critically evaluated. Though treatments such as corticosteroids have been commonly used with reported success for years, only rarely have AA treatments been thoroughly evaluated in randomized controlled trials; these trials have often been limited in scope and have demonstrated little benefit [3]. Notably, intralesional corticosteroid treatment, one of the most commonly used AA treatments [4], has never been evaluated in a randomized controlled trial [3].

In the absence of a strong evidence base, treatment guidelines that stratify patients by age and disease extent have been proposed [5-7]. These guidelines recommend intralesional steroids as first line therapy for patch hair loss in adults. They generally do not recommend painful or more aggressive treatments for children under 10. Because alopecia totalis (AT) and universalis (AU) are more difficult to treat than patch hair loss, they suggest that physicians consider the option of no medical treatment. It is important to note that the patient's desire for treatment of this disorder cannot be overestimated.

Without firm treatment guidelines, the current AA treatment practices of dermatologists vary greatly. The 1990-2000 National Ambulatory Medical Care Survey (NAMCS) indicated that topical and intralesional triamcinolone were the most commonly prescribed AA treatments, followed by augmented betamethasone propionate and minoxidil [4]. However, the NAMCS results do not provide the most commonly used AA treatments stratified by patient age or disease severity. The current practices in treatment of children compared to adults and of progressive stages of hair loss remain unknown.

The aim of this study was to examine the current practices of southeastern United States dermatologists for the treatment of AA. We surveyed dermatologists in the region by mailed questionnaire, and we compared dermatologist-reported treatment preferences to patient-reported treatment data obtained from the National Alopecia Areata Registry. Reported in this article are the results of our survey and subsequent analyses.

## Methods

This study was approved by the Institutional Review Board of the University of North Carolina (UNC) School of Medicine (Study #07-1872, approved November 13, 2007).

### Survey Administration

A mailing list of 1888 dermatologists in the southeastern United States (Virginia, North Carolina, South Carolina, Georgia, and Florida) was obtained from the American Academy of Dermatology (December 3, 2007). The list included all members of the Academy, including those with military addresses, in the region. In January 2008, dermatologists were sent anonymous surveys regarding their preferences for treatment of AA and a cover letter by the investigators by U.S. Postal Service. Postage-paid envelopes were included for responses. Topics included:

• Frequency of recommending any medical treatment (choices: all/most/some/none of the time) for children/adults with first episode patch hair loss/multiple episodes patch loss/alopecia totalis (AT)/alopecia universalis (AU),

• Choice of treatment for children/adults with first episode patch hair loss/multiple episodes patch loss/AT/AU,

• Use of wigs and eyebrow tattoos,

• Barriers to use of individual treatments, and

• Physician demographics.

Children were defined as "preadolescents" in the survey, which was developed by the authors and was piloted and evaluated for clarity with UNC-Chapel Hill dermatology residents. A copy of the survey is included as a supplement to this article [see Additional file 1].

Surveys were not sent to the UNC-Chapel Hill Dermatology faculty. Surveys completed by physicians who indicated that they did not practice general dermatology in the United States were excluded from analysis.

### Analyses of Survey Data

For the purpose of analysis of the survey data, it was assumed that first episode of patch hair loss, multiple episodes of patch hair loss, AT and AU are forms of the same condition with increasing degrees of severity.

For analysis of frequency of treatment, Wilcoxon signed-ranks test procedures were used when comparing respondents' treatment of children compared to adults, and Friedman test procedures (nonparametric repeated measures comparisons) were used when comparing respondents' treatment of the four AA stages of disease. Each Friedman test was followed by pairwise comparisons of disease stages using Dunn's multiple comparisons test. For analysis of frequency of individual medication usage, McNemar's test was used when comparing respondents' treatment of children compared to adults. In the application of these test procedures, respondents who had answered with "Not Applicable (I do not see this kind of patient)" for one or more of the groups being compared were excluded from the analysis.

Exploratory analyses using Mann-Whitney tests were performed to investigate whether certain demographic characteristics of the respondents were predictive of the frequencies of treatments.

All statistical hypothesis tests were two-sided. The p-values obtained were used for purposes of generating hypotheses and should not be treated as confirmatory. Because the purpose of analysis was hypothesis generation rather than hypothesis confirmation, adjusting for multiple comparisons was not necessary.

### National Alopecia Areata Registry (NAAR) Data

Patient treatment histories retrieved from the National Alopecia Areata Registry http://www.mdanderson.org/departments/alopecia/ served as a complementary second source of information. Patients who donated DNA to the registry completed a "long form" that recorded their current and previous treatments as well as disease classification. Patients were classified by overall (lifelong) disease severity into four categories: transient alopecia areata (AAT), patchy alopecia areata (AAP), alopecia totalis (AT), and alopecia universalis (AU). Within each AA category, the registry database was analyzed for number of instances in which a patient had previously received or was currently receiving a certain treatment. A total of 1264 patients had completed the long form at the time when the database was analyzed.

### Analyses of NAAR Data

Within each disease stage, the number of database mentions of a particular drug category was divided by the number of patients with that stage of disease to produce a "drug category mentions per patient" ratio.

For each category of medications, the number of mentions per patient was assumed to follow a Poisson distribution in each of the patient strata defined by disease stage. In the special case of the medication category "None", we assumed that each patient can contribute to the count of mentions of "None" no more than one time. Consequently, a binomial distribution was assumed for this special category. Under these assumptions, the summary totals available from the registry were sufficient for the fitting of the desired generalized log-linear model for Poisson regression and for fitting the desired binomial model. These models were fitted separately for each category of medication (11 separate models). For each, the results of fitting the model were used to test the overall null hypothesis "no difference among the 4 disease-stage strata". For the overall tests that were statistically significant at level α = 0.005, pairwise comparisons between strata were examined and statistical tests of size α = 0.01 were performed. Thus, a difference between two strata for a given category of medication was considered statistically significant if both the overall test p-value was smaller than 0.005 and the pairwise test p-value was smaller than 0.01.

## Results

### Survey

Of the 1870 surveys mailed, 313 surveys (16.7%) were returned. Out of those, 44 surveys were excluded from further analysis because they were completed by retired dermatologists, administrators, or non-practicing physicians. The remaining 269 surveys were completed by respondents who indicated that they practiced general dermatology in the United States at the time of survey. Thus, the response rate from this target population was 14.7% (269/1826).

Respondents recommended medical treatment in adults more frequently than in children [see Additional file 2: **Table S1**]. This tendency was notably evident for patch hair loss (first or multiple episodes) and weakly evident for AT and AU. For both adults and children, comparisons of disease stages showed that multiple episodes patch hair loss was treated more frequently than AT (results of Friedman tests not shown).

Respondents' choices of medications for treatment of children and adults were notable for increased systemic corticosteroid usage with increasing disease severity and frequent usage of topical and intralesional corticosteroids, anthralin, and minoxidil [see Additional file 3: **Table S2**]. Topical and intralesional steroids were used more frequently for multiple episodes patch hair loss than AT, while systemic corticosteroids were used less frequently for multiple episodes patchy hair loss than AT [see Additional file 3: **Table S2**].

Usage of the most commonly recommended medications (topical, intralesional, and systemic corticosteroids and minoxidil) was evaluated in children compared to adults. Topical corticosteroids were used more frequently for patch hair loss in children than adults; intralesional and systemic corticosteroids and minoxidil were used more frequently in adults than children for all four disease stages [see Additional file 4: **Table S3**].

In a free response box on the survey, respondents listed other treatments that they used for AA that were not provided as response options (Table 1). The most commonly mentioned treatments were calcineurin inhibitors (73 mentions).

**Table 1:** Other medications used for treatment of alopecia areata.

Treatment	Number of mentions
Protopic (tacrolimus)	41

Elidel (pimecrolimus)	32

Aldara (imiquimod)	16

Cyclosporine	10

Retinoids	6

Azulfidine (sulfasalazine)	4

Amevive (alefacept)	3

Biotin	2

Dovonex (calcipotriene)	2

XTRAC laser	2

Atarax	1

Cryo spray	1

DMSO (dimethylsulfoxide?)	1

Diphencyprone**	1

Enbrel (etanercept)	1

Metronidazole	1

Multivitamin	1

Nioxin Hair Care System*	1

Rogaine (minoxidil)	1

Rogaine with Tazorac gel	1

Vytorin	1

*Does not contain any drugs, but cited by one respondent

**Respondent used in past, but now cannot obtain it.

Of those respondents who reported seeing scalp hair loss in their practice (N = 260), 12% recommended wigs all or most of the time; 80% some of the time; and 6% none of the time. Of those who reported seeing eyebrow loss (N = 239), 7% recommended temporary or permanent eyebrow tattoos all or most of the time; 45% some of the time; and 48% none of the time.

Among the most commonly cited barriers to the use of various treatments of AA were the risk of side effects when using systemic corticosteroids and methotrexate, pain associated with intralesional corticosteroids, and time commitment and cost of PUVA/UVB therapy [see Additional file 5: **Table S4**]. Other frequently cited barriers were patient age when using intralesional corticosteroids and lack of experience with topical immunotherapy and methotrexate. Lack of supporting evidence and non-FDA approval were not commonly cited as barriers to any treatment.

Most respondents indicated that they did not practice a dermatologic subspecialty, and a large number of respondents saw less than two new AA patients monthly (of which 25% or less were children) and practiced in suburban areas (Table 2). Earlier in the analysis, AT was found to be the "critical point" of disease severity at which treatment frequency began to drop; thus, certain demographic divisions, specifically male versus female gender, 0-2 versus 3 or more new AA patients per month, and 0-5 versus 6 or more years in practice, were compared in terms of frequency of treatment of AT in adults. No differences were found in frequency of treatment of AT between these demographic divisions (data not shown). Additionally, no substantial differences were found in frequency of treatment of AT in children in those practices in which ≤ 25% and > 25% of new AA patients were children.

**Table 2:** Respondent demographics (N = 253; not all respondents answered demographics questions)

New AA pts per month	0 to 2	63.6%
	
	3 to 5	29.6%
	
	6 to 10	5.5%
	
	11 to 20	0.4%
	
	20+	0.8%
**Percent of new AA pts that are children**	None	11.5%
	
	1 to 25	66.4%
	
	26 to 50	17.8%
	
	51 to 75	3.6%
	
	76 to 99	0.4%
	
	All	0.4%

**Subspecialty certification**	None	88.5%
	
	Pediatric	1.2%
	
	Procedural	5.1%
	
	Other	5.1%

**Practice setting**	Solo	36.0%
	
	Group derm only	50.2%
	
	Multispecialty	6.3%
	
	Academic	5.9%
	
	Government	1.6%

**Years in practice (N = 251)**	0 to 5	18.3%
	
	6 to 10	15.1%
	
	11 to 20	26.3%
	
	21+	40.2%

**Gender (N = 252)**	Male	59.9%
	
	Female	40.1%

**Location**	Urban	31.2%
	
	Suburban	58.5%
	
	Rural	6.7%
	
	Other	3.6%

### National Alopecia Areata Registry

**Figure 1:**
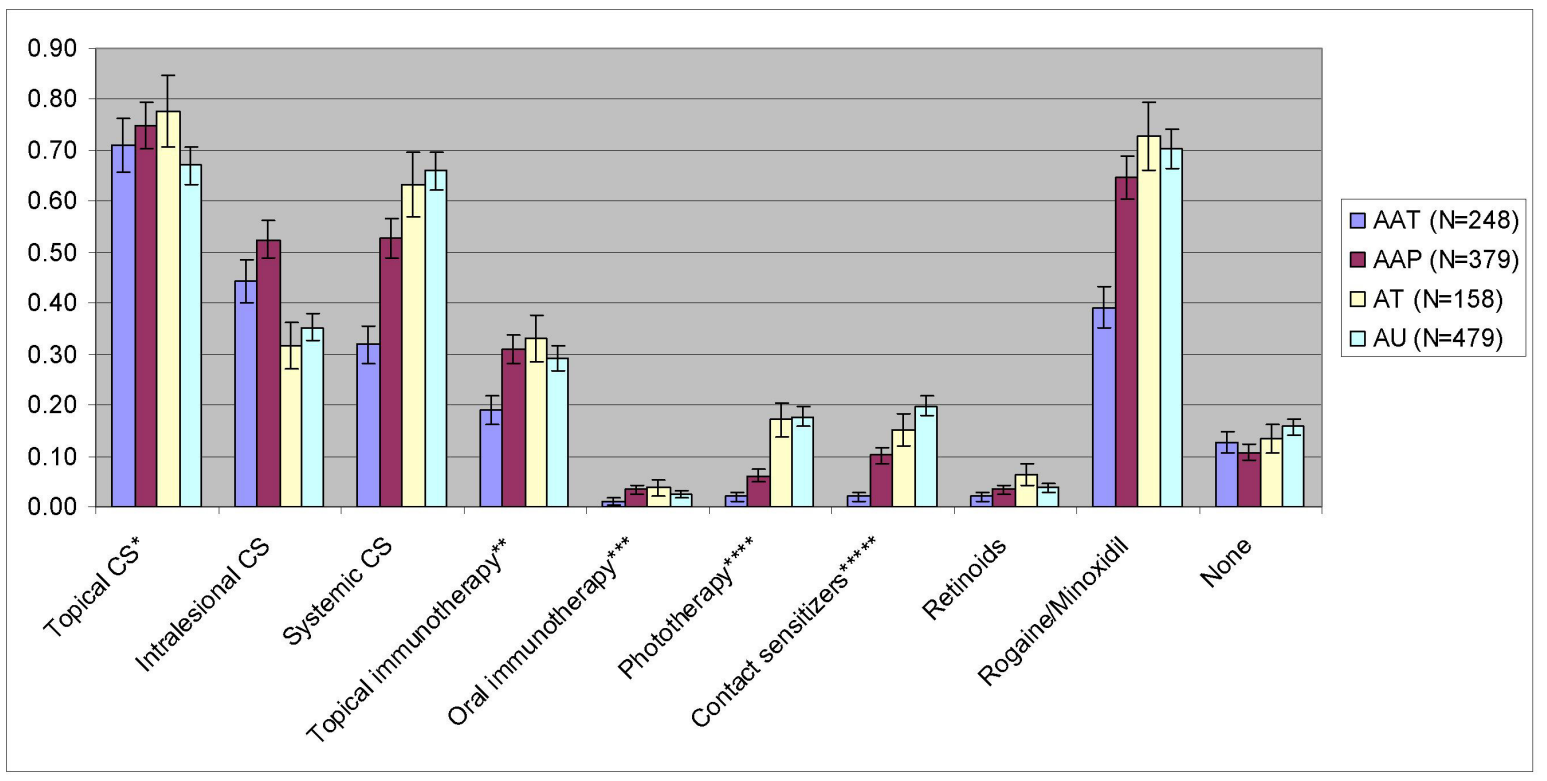
**Drugs received by patients registered in the National Alopecia Areata Registry**. Values shown are mention rates with approximate 95% confidence intervals. *CS = corticosteroids. ** FK506/cyclosporine/imiquimod/pimecrolimus/other. ***FK506/cyclosporine/other. ****PUVA/UVB. *****DNCB Dinitrochlorobenzene/diphencyprone/squaric acid dibutylester

Treatment data from the NAAR showed that over 10% of patients in each AA disease category reported having received no treatment over the course of their disease (Figure 1). Though there was a trend towards AU patients receiving no treatment most often (15.7%), there was no statistically significant difference between disease stages in the percentage of patients receiving no treatment (p = 0.1759).

Of the drug categories tested, the "mentions per patient" ratios of systemic corticosteroids, topical immunotherapy, phototherapy, contact sensitizers, and minoxidil were significantly different between patients with transient AA and patchy AA [see Additional file 6:**Table S5**]. The "mentions per patient" ratios of intralesional corticosteroids and phototherapy were significantly different between patients with patchy AA and AT [see Additional file 6: **Table S5**]. No drug category differed in use between patients with AT and AU.

## Discussion

Our primary aim was to study current AA treatment practices across age groups and stages of progressive disease severity. Our results suggest that children receive treatment less frequently than adults. The most commonly used AA treatments overall are corticosteroids and minoxidil. The frequency of AA treatment decreases with progressive disease severity, and AT appears to be the "critical point" at which there is a drop in rates of certain treatments.

Our survey respondents were largely general dermatologists in suburban, solo/group private practice. The gender distribution of respondents was 60% male/40% female. Most respondents saw five or fewer new AA patients per month. Interestingly, the majority of our respondents had been in practice for over 10 years, and a relatively high number (40%) had practiced for 21 years or more. The American Academy of Dermatology (AAD) reported the gender distribution of its members in 2007 to be 62% male/38% female, and the 2007 AAD Practice Profile Survey (sponsored by the AAD) reported the practice settings of a random sample of AAD members practicing dermatology in the U.S. to be 54.8% suburban and 44.2% solo practice/33.1% dermatology group practice. Thus, the demographics of our study's respondents appear to be generally consistent with the overall demographics of U.S. dermatologists.

We observed that respondents treated children less frequently than adults, particularly in cases of patch hair loss. Dermatologists may be less willing to treat children due to the pain or risk associated with treatment [6]. They may also seek to avert the patient's attention from hair loss or to avoid social disruption [5], or to prevent a "roller coaster of improvements and setbacks" in the absence of proven treatments [8]. As emphasized by a recent review, the primary limitation in evidence-based treatment of children with AA is the lack of randomized controlled trials of AA therapies [9]. AA is a disease with strong potential for psychological trauma, particularly in children [10,11], and it is important to optimize treatment in this age group. Thus, some recommend that pediatric patients receive counseling or join a support group from the onset of the disease [8]. Our survey did not assess how often physicians recommend support groups or counseling to their patients with AA; such an assessment would be worthwhile in the future.

Our observation that the frequency of AA treatment decreases with progressive disease severity may similarly reflect physician reluctance to expose patients to the risks, costs, and side effects of treatment in the absence of proven options. It is interesting to note that while the number of respondents prescribing no treatment increases with disease severity, so does the number of respondents prescribing systemic drugs with more side effects such as systemic corticosteroids and methotrexate. This suggests that two different approaches to treatment exist among dermatologists - some see a greater need to treat with more widespread disease, while others see less value in treatment.

Topical and intralesional corticosteroids and minoxidil were the most commonly used treatments cited by our respondents; however, while these drugs have frequently been reported to be useful and successful in management of AA, they are of questionable long-term and overall benefit. A recent systematic review found no randomized, controlled trials of intralesional corticosteroids, systemic corticosteroids, or minoxidil that demonstrated clinically significant hair regrowth [3]. The meta-analysis identified only one trial that showed a statistically significant difference between hair regrowth with betamethasone valerate foam and betamethasone dipropionate lotion; the study did not include a vehicle-control arm [12]. As the authors noted, corticosteroids and minoxidil appear to be in wide use due to their relative safety, but their use remains unsupported by rigorous evidence. We noted that these drugs are used quite frequently even to treat more severe forms of AA (AT/AU) per our survey results, though many perceive them to be ineffective (Table 6) - again, we posit that their relative safety and the physician's desire to offer treatment leads to their use despite lack of evidence as to their efficacy.

We noted that use of wigs and eyebrow tattoos for scalp and eyebrow hair loss is limited. As it is very important to AA patients to optimize their cosmetic appearance, more widespread use of wigs and tattoos may be warranted.

The most commonly cited barriers to the use of various treatments were risk of side effects; pain associated with treatment; excessive time commitment, compliance or cost; and lack of experience with the treatment. Interestingly, lack of evidence and FDA approval were not commonly cited as barriers to the use of treatment. Our findings suggest that many physicians desire to treat despite the limited amount of available evidence, but that many treatments are simply unsatisfactory or unfamiliar.

Data from the NAAR showed that, as suggested in our survey findings, a relatively high percentage of AA patients never receive treatment even in the more advanced stages of AA. NAAR data did not reveal any consistent pattern of drug use. Even the most commonly employed treatments (topical, intralesional, and systemic corticosteroids, topical immunotherapy, and minoxidil) show no consistent pattern of increasing or decreasing use with progressive stages of AA.

Limitations of our survey study include our response rate, which was 14.6% of our target population. It is not known whether the respondents to our survey are a representative sample of the target population. Thus, substantial "survey bias" is possible, and it is reasonable to believe that some degree of "survey bias" is likely to be present. We used a paper questionnaire rather than a web-based survey format because dermatologists' e-mail addresses for an entire region could not be obtained; our choice of format may have skewed the demographics of our responders. We simply defined children as "preadolescents" in our questionnaire, leaving determination of an age cutoff to the respondent; some respondents may have defined the "preadolescent" age range differently than others. Our survey instrument divided AA into four discrete stages (first episode of patch loss vs. multiple episodes of patch loss vs. AT vs. AU) in order to simplify and categorize physicians' treatment preferences, but within each disease stage lies a spectrum of disease presentations with varied prognoses that may warrant different treatment approaches; these nuances may have been lost in our study. Our survey did not ask physicians about their diagnostic and treatment approaches for related medical conditions such as thyroid disease.

The NAAR portion of our study was limited by our use of the "mentions per patient" ratio; because we could not access individual patient treatment histories, we could not calculate percentages of patients who had used treatments from a certain drug category (as we could not account for cases in which a patient had received two or more drugs from the same category). However, we were able to determine the percentages of patients who had received no treatment over the course of their disease. The experiences of the patients in the NAAR database may not be representative of the experiences of all AA patients.

Our survey relied on physician recall of practice habits; medical records were not examined for verification of physician practices. Nonetheless, our physician-reported findings showed good congruence with patient-reported treatment data from the NAAR.

## Conclusion

Analyses of both our survey data and patient-reported treatment data from the NAAR lead to the conclusion that dermatologists' treatment of pediatric and adult patients and of progressive disease stages is inconsistent. Younger patients and those with more severe disease appear to be less likely to receive treatment. The acquisition of a stronger evidence base and the development of new treatments will provide better and more consistently employed treatment options.

## Abbreviations

Abbreviations used in this report are as follows: AA: Alopecia areata; AAP: Patchy alopecia areata; AAT: Transient alopecia areata; AT: Alopecia totalis; AU: Alopecia universalis; FDA: Food and Drug Administration; N/A: Not applicable; NAAR: National alopecia areata registry; PUVA: Psoralen plus UVA treatment

## Competing interests

The authors declare that they have no competing interests.

## Authors' contributions

NM participated in the design of the study, administration of the survey, statistical analysis, and manuscript preparation. DSM participated in the design of the study, statistical analysis, and manuscript preparation. MD (representing the National Alopecia Areata Registry) gathered and supplied patient data from the Registry. PWS participated in the statistical analysis. LAG participated in the design of the study, statistical analysis, and manuscript preparation. All authors read and approved the final manuscript.

## Pre-publication history

The pre-publication history for this paper can be accessed here:

http://www.biomedcentral.com/1471-5945/9/11/prepub

## Supplementary Material

Additional file 1 Survey instrument used in this study. Click here for file

Additional file 2 **Table S1**. Frequency of recommended treatment in preadolescents compared to adolescents and adults with progressive stages of alopecia areata. Click here for file

Additional file 3 **Table S2**. Percentage of respondents that recommend various drugs for treatment of progressive stages of alopecia areata. Click here for file

Additional file 4 **Table S3**. Use of topical, intralesional, and systemic corticosteroids and minoxidil in children versus adults. Click here for file

Additional file 5 **Table S4**. Barriers to the use of various alopecia areata treatments. Click here for file

Additional file 6 **Table S5**. "Mentions per patient" ratio for each drug category over four stages of disease: transient alopecia areata, patchy alopecia areata, alopecia totalis, and alopecia universalis. Click here for file
