# Supplementary material for: Attitudes of dermatologists in the southeastern United States regarding treatment of alopecia areata: a cross-sectional survey study
Source: BMC Dermatol. 2009 Nov 12;9:11. doi: 10.1186/1471-5945-9-11 (PMC2789708; doi:10.1186/1471-5945-9-11)
Supplement: Additional file 1 — Survey instrument used in this study. [file 1471-5945-9-11-S1.pdf]

Questions Regarding Treatment of Alopecia Areata

If you do not actively practice general dermatology in the United States, please:	☐	Retired
1. indicate why not by checking all appropriate boxes to the right	☐	Administrator
2. return this questionnaire in the enclosed stamped envelope. Thank you.	☐	Other ______________________________

We are interested in the current practices of dermatologists in treatment of alopecia areata in the southeastern United States. The following questions pertain to your preferred methods of treating alopecia areata in children and adults. For some questions, only one answer should be marked, while for others, multiple answers can be marked as indicated. Please also note that some questions may be answered with N/A (not applicable).

1. How frequently do you recommend any medical treatment at all for: *(mark one answer for each row)*

		Portion of the time				N/A—I do not see this in my practice
		All	Most	Some	None	
**Preadolescent children with:**	**First episode of patch hair loss?**	☐	☐	☐	☐	☐
	**Multiple episodes of patch hair loss?**	☐	☐	☐	☐	☐
	**Loss of all scalp hair (alopecia totalis)?**	☐	☐	☐	☐	☐
	**Loss of all scalp and body hair (alopecia universalis)?**	☐	☐	☐	☐	☐
**Adolescents and adults with:**	**First episode of patch hair loss?**	☐	☐	☐	☐	☐
	**Multiple episodes of patch hair loss?**	☐	☐	☐	☐	☐
	**Loss of all scalp hair (alopecia totalis)?**	☐	☐	☐	☐	☐
	**Loss of all scalp and body hair (alopecia universalis)?**	☐	☐	☐	☐	☐

2. Which treatment(s)*, if any, do you use as medical treatment for: *(mark all that apply for each row)*

	I do not recommend any medical treatment	Topical corticosteroids	Intralesional corticosteroids	Systemic corticosteroids	Anthralin	Minoxidil	Psoralen plus ultraviolet A or narrow band ultraviolet B light therapy	Topical immunotherapy (squaric acid dibutylester or 2,3-diphenylcyclopro-penone)	Methotrexate	N/A—I do not see this in my practice
**Preadolescents with:**										
**Patch hair loss (first episode)?**	☐	☐	☐	☐	☐	☐	☐	☐	☐	☐
**Patch hair loss (multiple episodes)?**	☐	☐	☐	☐	☐	☐	☐	☐	☐	☐
**Loss of all scalp hair (AT)?**	☐	☐	☐	☐	☐	☐	☐	☐	☐	☐
**Loss of all scalp and body hair (AU)?**	☐	☐	☐	☐	☐	☐	☐	☐	☐	☐
**Adolescents and adults with:**										
**Patch hair loss (first episode)?**	☐	☐	☐	☐	☐	☐	☐	☐	☐	☐
**Patch hair loss (multiple episodes)?**	☐	☐	☐	☐	☐	☐	☐	☐	☐	☐
**Loss of all scalp hair (AT)?**	☐	☐	☐	☐	☐	☐	☐	☐	☐	☐
**Loss of all scalp and body hair (AU)?**	☐	☐	☐	☐	☐	☐	☐	☐	☐	☐

*Please note that some of the treatments listed here are not FDA-approved for treatment of alopecia areata.

3. Other than those treatments listed as options in question 2, are there any other medical treatments that you have used or currently use for alopecia areata? *(If so, please list)* ____________________

4. How often do you recommend: *(mark one answer for each row)*

	Portion of the time				
	All	Most	Some	None	N/A—I do not see this in my
**Wigs or hairpieces for scalp hair loss?**	☐	☐	☐	☐	☐
**Temporary or permanent tattooing for eyebrow loss?**	☐	☐	☐	☐	☐

5. Are you likely to recommend the following alopecia areata treatments* to patients who are potential candidates for them? *(mark one answer for each row)*

	Yes, in most cases	Yes, in some cases	Yes, but rarely	Never
**No treatment**	☐	☐	☐	☐
**Topical corticosteroids**	☐	☐	☐	☐
**Intralesional corticosteroids**	☐	☐	☐	☐
**Systemic corticosteroids**	☐	☐	☐	☐
**Anthralin**	☐	☐	☐	☐
**Minoxidil**	☐	☐	☐	☐
**Psoralen plus ultraviolet A or narrow-band UVB light therapy**	☐	☐	☐	☐
**Topical immunotherapy (squaric acid dibutylester or 2,3-diphenylcyclopropenone)**	☐	☐	☐	☐
**Methotrexate**	☐	☐	☐	☐

*Please note that some of the treatments listed here are not FDA-approved for treatment of alopecia areata.

6. What are the barriers to your use of the following treatments*? *(mark all that apply for each row)*

	No barriers	Risk of side effects	Painful or difficult to tolerate	Patient age	Ineffective	Requires excessive time commitment, compliance, and/or cost	Patient concerns	Lack of experience with this treatment	Lack of evidence to support use of this treatment	Not FDA-approved for treatment of alopecia areata
**Topical corticosteroids**	☐	☐	☐	☐	☐	☐	☐	☐	☐	☐
**Intralesional corticosteroids**	☐	☐	☐	☐	☐	☐	☐	☐	☐	☐
**Systemic corticosteroids**	☐	☐	☐	☐	☐	☐	☐	☐	☐	☐
**Anthralin**	☐	☐	☐	☐	☐	☐	☐	☐	☐	☐
**Minoxidil**	☐	☐	☐	☐	☐	☐	☐	☐	☐	☐
**Psoralen plus ultraviolet A or narrow-band UVB light therapy**	☐	☐	☐	☐	☐	☐	☐	☐	☐	☐
**Topical immunotherapy (squaric acid dibutylester or 2,3-diphenylcyclo-propenone)**	☐	☐	☐	☐	☐	☐	☐	☐	☐	☐
**Methotrexate**	☐	☐	☐	☐	☐	☐	☐	☐	☐	☐

*Please note that some treatments listed here are not FDA-approved for treatment of alopecia areata.

7. How many new patients with alopecia areata do you see per month?

0-2	3-5	6-10	11-20	20+
☐	☐	☐	☐	☐

8. Of your patients with alopecia areata, how many are children?

None	1-25%	26-50%	51-75%	76-99%	All
☐	☐	☐	☐	☐	☐

9. Are you certified in any of the following subspecialties?

Pediatric dermatology	Procedural dermatology	Other ___________________
☐	☐	☐

10. How would you describe your current practice setting?

Solo practice	Group practice—dermatology only	Multispecialty practice	Academic	Government
☐	☐	☐	☐	☐

11. For how many years have you been in practice?

0-5	6-10	11-20	21+
☐	☐	☐	☐

12. Check the appropriate box indicating your gender.

Male	Female
☐	☐

13. Check the appropriate box describing your practice.

Urban	Suburban	Rural	Other________________
☐	☐	☐	☐
