# Supplementary material for: Attitudes of dermatologists in the southeastern United States regarding treatment of alopecia areata: a cross-sectional survey study
Source: BMC Dermatol. 2009 Nov 12;9:11. doi: 10.1186/1471-5945-9-11 (PMC2789708; doi:10.1186/1471-5945-9-11)
Supplement: Additional file 2 — Table S1. Frequency of recommended treatment in preadolescents compared to adolescents and adults with progressive stages of alopecia areata. [file 1471-5945-9-11-S2.docx]

**Table 1: Frequency of recommended treatment in preadolescents compared to adolescents and adults with progressive stages of alopecia areata.**

|  |  | **Frequency of recommending any medical treatment**  Percentage of respondents | | | | **Wilcoxon Signed-Rank Test:  Children vs. Adults** |
| --- | --- | --- | --- | --- | --- | --- |
|  |  | *All of the time* | *Most of the time* | *Some of the time* | *Never* |  |
| **First episode patch hair loss**  (N = 254*) | Children | 50.0% | 34.6% | 12.6% | 2.8% | p (2-tail) = <0.0001 |
|  | Adults | 66.1% | 28.0% | 5.5% | 0.4% |  |
| **Multiple  episodes patch hair loss**  (N = 253*) | Children | 64.0% | 29.2% | 6.3% | 0.4% | p (2-tail) = <0.0001 |
|  | Adults | 74.7% | 22.9% | 2.4% | 0% |  |
| **Alopecia totalis**  (N = 186*) | Children | 55.4% | 15.1% | 22.6% | 7.0% | p (2-tail) = 0.101 |
|  | Adults | 58.6% | 14.0% | 21.5% | 5.9% |  |
| **Alopecia  universalis**  (N = 163*) | Children | 52.8% | 9.2% | 23.3% | 14.7% | p (2-tail) = 0.0629 |
|  | Adults | 57.1% | 8.0% | 20.2% | 14.7% |  |

Children with patch hair loss (first or multiple episodes) are treated less frequently than adults with patch hair loss (statistically significant).

*For each disease stage, respondents who answered “N/A – Do not see this in my practice” in regards to preadolescents and/or adolescents and adults are excluded.
