# Supplementary material for: Attitudes of dermatologists in the southeastern United States regarding treatment of alopecia areata: a cross-sectional survey study
Source: BMC Dermatol. 2009 Nov 12;9:11. doi: 10.1186/1471-5945-9-11 (PMC2789708; doi:10.1186/1471-5945-9-11)
Supplement: Additional file 3 — Table S2. Percentage of respondents that recommend various drugs for treatment of progressive stages of alopecia areata. [file 1471-5945-9-11-S3.docx]

**Table 2: Percentage of respondents that recommend various drugs for treatment of progressive stages of alopecia areata.**

|  | Number of respondents (excludes “N/A”  responses) | **Do not  recom-mend any medical treat-ment*** | **Topical  cortico-steroids** | **Intra-lesional  cortico-steroids** | **Systemic cortico-steroids** | **Anthralin** | **Minoxidil** | **PUVA/  narrow band UVB  therapy** | **Topical  immuno-therapy*** | **Metho-trexate** |
| --- | --- | --- | --- | --- | --- | --- | --- | --- | --- | --- |
| **First episode patch hair loss** | Children N=253 | 2.8% | 93.7% | 45.5% | 2.8% | 15.0% | 16.6% | 0.4% | 4.3% | 0 |
|  | Adults N=266 | 1.5% | 86.5% | 86.1% | 7.5% | 18.0% | 28.6% | 0.8% | 6.0% | 0 |
| **Multiple episodes patch hair loss** | Children N=252 | 1.2% | 91.3% | 64.3% | 12.3% | 25.8% | 24.2% | 2.0% | 9.1% | 0 |
|  | Adults N=266 | 1.5% | 83.8% | 95.1% | 27.8% | 30.8% | 42.1% | 3.8% | 13.9% | 0.8% |
| **Alopecia totalis** | Children N=171 | 9.9% | 55.6% | 22.8% | 49.7% | 24.0% | 24.0% | 15.2% | 18.7% | 2.9% |
|  | Adults N=199 | 5.5% | 53.8% | 34.7% | 61.3% | 30.2% | 37.2% | 20.1% | 27.1% | 7.0% |
| **Alopecia universalis** | Children N=145 | 22.8% | 35.9% | 17.9% | 47.6% | 13.8% | 14.5% | 16.6% | 13.8% | 3.4% |
|  | Adults N=164 | 20.7% | 34.1% | 23.2% | 53.7% | 16.5% | 23.8% | 21.3% | 20.7% | 9.1% |

Numbers of respondents who do not recommend any medical treatment in Table 2 does not exactly correspond with numbers who recommend treatment none of the time in Table 1. This is because selecting the “do not recommend any medical treatment” option in Table 2 did not preclude selecting other treatments as well if they are used for some patients.

*squaric acid dibutylester or 2,3-diphenylcyclopropenone
