# Supplementary material for: Attitudes of dermatologists in the southeastern United States regarding treatment of alopecia areata: a cross-sectional survey study
Source: BMC Dermatol. 2009 Nov 12;9:11. doi: 10.1186/1471-5945-9-11 (PMC2789708; doi:10.1186/1471-5945-9-11)
Supplement: Additional file 4 — Table S3. Use of topical, intralesional, and systemic corticosteroids and minoxidil in children versus adults. [file 1471-5945-9-11-S4.docx]

**Table 3: Use of topical, intralesional, and systemic corticosteroids and minoxidil in children versus adults: results of McNemar tests.**

|  | **Topical corticosteroids** | **Intralesional corticosteroids** | **Systemic corticosteroids** | **Minoxidil** |
| --- | --- | --- | --- | --- |
| **First episode patch hair loss** N = 253 | P_children_ = 0.937  P_adults_ = 0.866  p = 0.001 | P_children_ = 0.458  P_adults_ = 0.869  p = <0.001 | P_children_ = 0.046  P_adults_ = 0.131  p = 0.002 | P_children_ = 0.166  P_adults_ = 0.281  p = <0.001 |
| **Multiple episodes patch hair loss** N = 252 | P_children_ = 0.912  P_adults_ = 0.861  p = 0.016 | P_children_ = 0.643  P_adults_ = 0.952  p = <0.001 | P_children_ = 0.123  P_adults_ = 0.282  p = <0.001 | P_children_ = 0.246  P_adults_ = 0.421  p = <0.001 |
| **Alopecia totalis**  N = 169 | P_children_ = 0.547  P_adults_ = 0.547  p = 0.814 | P_children_ = 0.231  P_adults_ = 0.355  p = <0.001 | P_children_ = 0.497  P_adults_ = 0.604  p = 0.002 | P_children_ = 0.231  P_adults_ = 0.391  p = <0.001 |
| **Alopecia universalis** N = 143 | P_children_ = 0.364  P_adults_ = 0.364  p = 0.773 | P_children_ = 0.182  P_adults_ = 0.245  p = 0.008 | P_children_ = 0.476  P_adults_ = 0.559  p = 0.006 | P_children_ = 0.147  P_adults_ = 0.245  p = 0.004 |

P_children_ = Proportion of respondents who use drug in children

P_adults_ = Proportion of respondents who use drug in adults

p = Two-tail p-value; p<0.05 suggests difference in P_children_ and P_adults_
