# Supplementary material for: Attitudes of dermatologists in the southeastern United States regarding treatment of alopecia areata: a cross-sectional survey study
Source: BMC Dermatol. 2009 Nov 12;9:11. doi: 10.1186/1471-5945-9-11 (PMC2789708; doi:10.1186/1471-5945-9-11)
Supplement: Additional file 5 — Table S4. Barriers to the use of various alopecia areata treatments. [file 1471-5945-9-11-S5.docx]

**Table 5: Barriers to the use of various alopecia areata treatments (N = 257).**

|  | **No barriers** | **Risk of side effects** | **Painful or difficult to tolerate** | **Patient age** | **Ineffective** | **Excessive time commitment, compliance or cost** | **Patient  concerns** | **Lack of  experience with  treatment** | **Lack of  evidence to support use of treatment** | **Not FDA-approved for  treatment of AA** |
| --- | --- | --- | --- | --- | --- | --- | --- | --- | --- | --- |
| **Topical  corticosteroids** | **58.8%** | **21.4%** | **0.8%** | **11.3%** | **22.6%** | **6.2%** | **8.2%** | **0%** | **2.7%** | **0%** |
| **Intralesional  corticosteroids** | **28.4%** | **26.1%** | **59.1%** | **44.0%** | **2.7%** | **3.9%** | **12.5%** | **0%** | **1.2%** | **0%** |
| **Systemic  corticosteroids** | **5.8%** | **87.9%** | **1.9%** | **29.2%** | **5.1%** | **2.3%** | **34.6%** | **3.5%** | **4.3%** | **1.6%** |
| **Anthralin** | **12.8%** | **17.5%** | **30.0%** | **5.8%** | **12.5%** | **31.1%** | **7.8%** | **25.3%** | **7.4%** | **1.6%** |
| **Minoxidil** | **38.1%** | **5.4%** | **0%** | **10.5%** | **29.6%** | **9.7%** | **3.1%** | **5.8%** | **14.0%** | **3.5%** |
| **Psoralen plus UVA/narrow band UVB** | **3.9%** | **23.3%** | **2.7%** | **14.0%** | **3.9%** | **50.6%** | **10.1%** | **37.4%** | **5.4%** | **4.3%** |
| **Topical  immunotherapy*** | **8.2%** | **26.1%** | **17.9%** | **9.7%** | **5.1%** | **27.2%** | **10.9%** | **44.7%** | **2.7%** | **11.3%** |
| **Methotrexate** | **1.9%** | **63.4%** | **2.3%** | **21.4%** | **2.3%** | **12.1%** | **23.7%** | **40.1%** | **15.6%** | **14.8%** |

*squaric acid dibutylester or 2,3-diphenylcyclopropenone
