# Supplementary material for: Attitudes of dermatologists in the southeastern United States regarding treatment of alopecia areata: a cross-sectional survey study
Source: BMC Dermatol. 2009 Nov 12;9:11. doi: 10.1186/1471-5945-9-11 (PMC2789708; doi:10.1186/1471-5945-9-11)
Supplement: Additional file 6 — Table S5. "Mentions per patient" ratio for each drug category over four stages of disease: transient alopecia areata, patchy alopecia areata, alopecia totalis, and alopecia universalis. [file 1471-5945-9-11-S6.docx]

**Table 7: “Mentions per patient” ratio for each drug category over four stages of disease: AAT (transient alopecia areata), AAP (patchy alopecia areata), AT (alopecia totalis), and AU (alopecia universalis).**

| **Drug category** | **Null hypothesis: No  differences between four stages of disease.**  **P-value:** | **Pairwise comparisons if differences exist:** | | | | | |
| --- | --- | --- | --- | --- | --- | --- | --- |
|  |  | **AAT vs. AAP** | | **AAP vs. AT** | | **AT vs. AU** | |
|  |  | Odds ratio | P-value | Odds ratio | P-value | Odds ratio | P-value |
| **Topical  corticosteroids** | 0.4125 | - | - | - | - | - | - |
| **Intralesional corticosteroids** | 0.0002 | 1.1838 | 0.1556 | **1.6592** | **0.0014** | 0.8969 | 0.4993 |
| **Systemic  corticosteroids** | <.0001 | **1.6566** | **0.0001** | 0.8338 | 0.1377 | 0.9594 | 0.7178 |
| **Topical immunotherapy** | 0.0129 | **1.6289** | **0.0047** | 0.9380 | 0.7009 | 1.1260 | 0.4648 |
| **Oral immunotherapy** | 0.2452 | - | - | - | - | - | - |
| **Phototherapy** | <.0001 | **3.0100** | **0.0255** | **0.3551** | **0.003** | 0.9630 | 0.8645 |
| **Contact sensitizers** | <.0001 | **4.9731** | **0.0007** | 0.6601 | 0.1111 | 0.7659 | 0.2430 |
| **Retinoids** | 0.1889 | - | - | - | - | - | - |
| **Minoxidil** | <.0001 | **1.6527** | **<0.0001** | 0.8881 | 0.1004 | 1.0345 | 0.7532 |
